# Supplementary material for: CDK1 dependent phosphorylation of hTERT contributes to cancer progression
Source: Nat Commun. 2020 Mar 25;11:1557. doi: 10.1038/s41467-020-15289-7 (PMC7096428; doi:10.1038/s41467-020-15289-7)
Supplement: Supplementary file 1 — Supplementary Information [file 41467_2020_15289_MOESM1_ESM.pdf]

Supplementary Information

CDK1 dependent phosphorylation of hTERT contributes to cancer progression

Yasukawa et al.

Supplementary Figures 1-20

Supplementary Tables 1-7

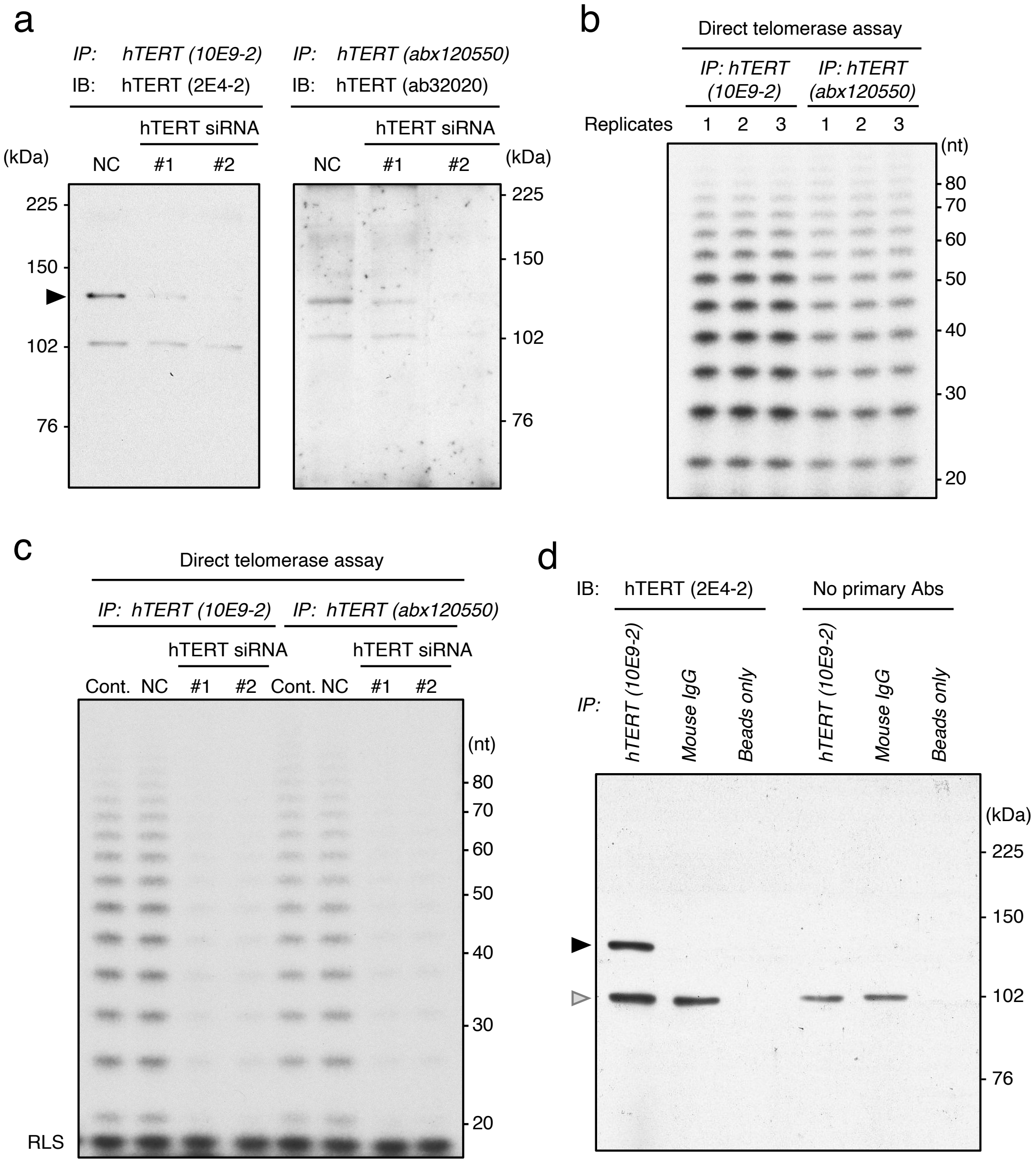
a
IP: hTERT (10E9-2)
IB: hTERT (2E4-2)
hTERT siRNA
(kDa)
NC
#1
#2
225
150
102
76
IP: hTERT (abx120550)
IB: hTERT (ab32020)
hTERT siRNA
NC
#1
#2
(kDa)
225
150
102
76
b
Direct telomerase assay
IP: hTERT (10E9-2)
IP: hTERT (abx120550)
Replicates
1 2 3 1 2 3
(nt)
80
70
60
50
40
30
20
c
Direct telomerase assay
IP: hTERT (10E9-2)
IP: hTERT (abx120550)
hTERT siRNA
hTERT siRNA
Cont. NC #1 #2 Cont. NC #1 #2
(nt)
80
70
60
50
40
30
20
RLS
d
IB: hTERT (2E4-2)
No primary Abs
IP:
hTERT (10E9-2)
Mouse IgG
Beads only
hTERT (10E9-2)
Mouse IgG
Beads only
(kDa)
225
150
102
76

Supplementary Figure 1 Confirmation of anti-hTERT antibodies used in the study

a, The endogenous hTERT proteins were immunoprecipitated with anti-hTERT mouse mAb (clone 10E9-2) or anti-hTERT sheep pAbs (abx120550) from HeLa cells transfected with two different siRNAs specific for hTERT or siNC followed by nocodazole treatment. The proteins were detected by anti-hTERT mouse mAb (clone 2E4-2) or anti-hTERT rabbit mAb (ab32020). **b,** Direct telomerase assay using hTERT proteins immunoprecipitated with anti-hTERT mouse mAb (clone 10E9-2) or anti-hTERT sheep pAbs (abx120550) from HeLa cells. **c,** Direct telomerase assay using hTERT proteins immunoprecipitated with anti-hTERT mouse mAb (clone 10E9-2) or anti-hTERT sheep pAbs (abx120550) from HeLa cells transfected with two different siRNAs specific for hTERT or siNC. RLS indicates recovery/loading standard. **d,** "No primary Abs" (without a reaction with primary antibody in the immunoblotting process) indicates that the band at 102 kDa (indicated by gray arrowhead) was nonspecific signal from the secondary antibody. In addition, "Mouse IgG" as an irrelevant monoclonal Ab for the immunoprecipitation process also recovers this 102 kDa band while "Beads only" failed to recover the band. We conclude that the band at 102 kDa is from a protein that is "nonspecifically" immunoprecipitated by a mouse IgG and "nonspecifically" detected by secondary antibody (anti-mouse IgG from rat). Experiments were repeated twice (for **a**, **b**, **c**) and three times (for **d**) with similar results. Source data are provided in the Source Data file.

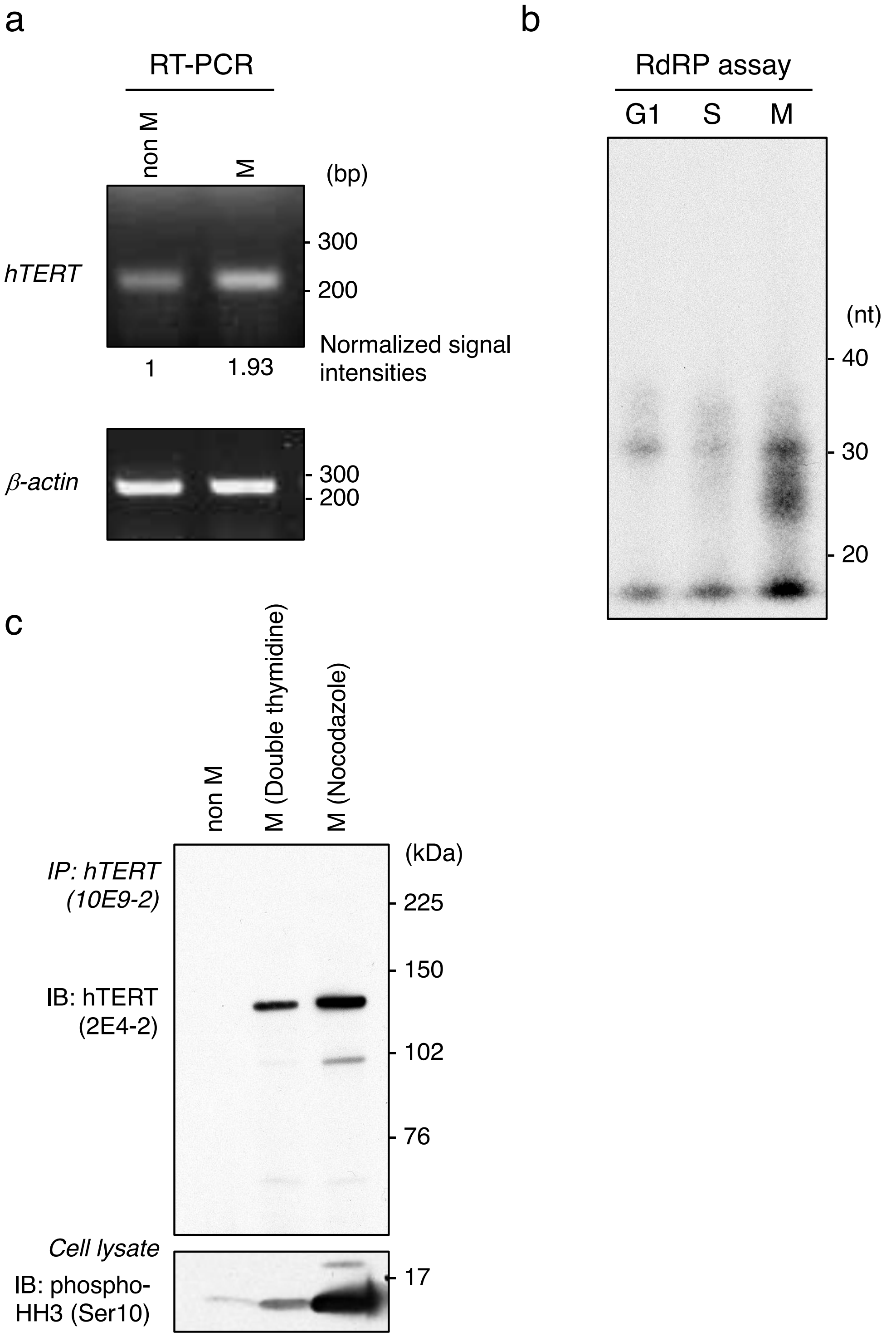
a
RT-PCR
non M
M
(bp)
hTERT
300
200
Normalized signal intensities
1
1.93
β-actin
300
200
b
RdRP assay
G1
S
M
(nt)
40
30
20
c
non M
M (Double thymidine)
M (Nocodazole)
(kDa)
IP: hTERT (10E9-2)
IB: hTERT (2E4-2)
225
150
102
76
Cell lysate
IB: phospho-HH3 (Ser10)
17

Supplementary Figure 2 Mitotic specific accumulation of hTERT in HeLa cells **a,** RT-PCR was performed to confirm the expression of *hTERT* mRNA in HeLa cells treated with DMSO (non M) or nocodazole (M). The normalized signal intensities with *β-actin* are noted below the panel. **b,** IP-RdRP assay using HeLa cells synchronized to G1-, S- and M- phase, respectively. **c,** HeLa cells were manipulated in mitosis by double thymidine block or nocodazole treatment. Cells arrested in mitosis were confirmed by anti-phospho-histone H3 (Ser10) antibodies. Experiments were repeated three times (for **a**) and twice (for **b**, **c**) with similar results. Source data are provided in the Source Data file.

hTERT_191-306

```
191                                206
SGPRRRLGCE  RAWNHSVREA  GVPLGLPAPG
                                      249
ARRRGGSASR  SLPLPKRPRR  GAAPEPERTP
                               274
VGQGSWAHPG  RTRGPSDRGF  CVVSPARPAE
  283                                306
EATSLEGALS  GTRHSHPSVG  RQHHAG
```

Supplementary Figure 3 Confirmation of phosphorylation site of hTERT_191-306 protein

The recombinant hTERT fragment proteins (hTERT_191-306) were phosphorylated by CDK1-cyclinB or IKK2_2-664 *in vitro* and analyzed by MS to confirm the phosphorylation sites. Threonine residue denoted with magenta letter was specifically phosphorylated by CDK1-cyclinB. Blue letter indicates threonine residue phosphorylated by IKK2_2-664 and green letters indicate serine residues phosphorylated by both CDK1-cyclinB and IKK2_2-664. See the details in **Supplementary Table 1**.

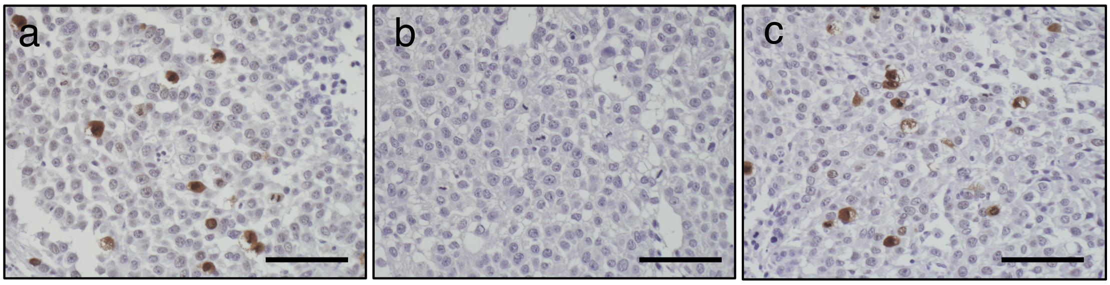

Supplementary Figure 4 Validation of the specificity of the anti-249T-P antibodies for IHC staining

a, Paraffin embedded sections of Huh7 xenografts were stained with anti-249T-P antibodies. **b** and **c,** Paraffin embedded sections of Huh7 xenografts were incubated with 0.4 μg/mL of phosphopeptides (**b**) or nonphosphopeptides (**c**) simultaneously with the same concentration of anti-249T-P pAbs. Scale bar: 100 μm.

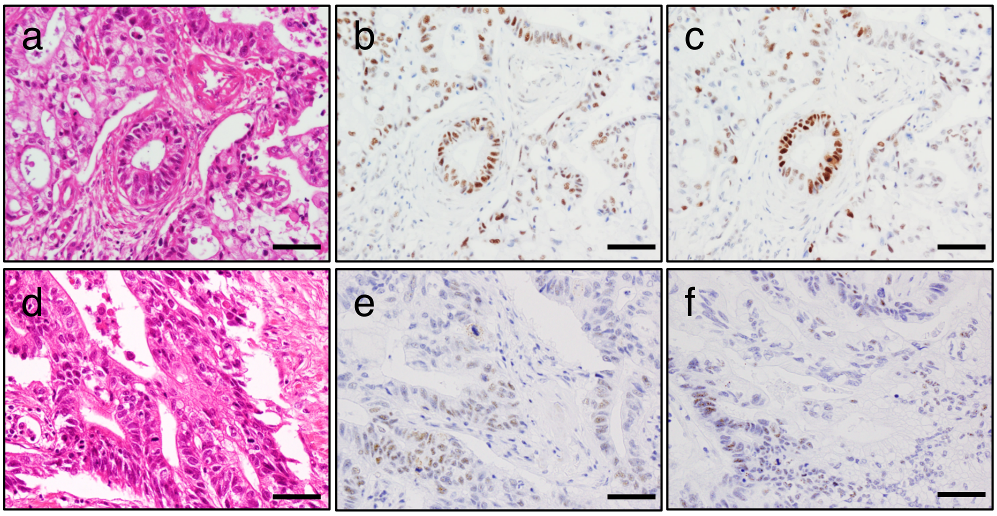

Supplementary Figure 5 Confirmation of the specificity of anti-249T-P and TpMab-1 antibodies

a-f, Hematoxilyn and elosin (HE) staining (**a** and **d**), IHC staining with anti-249T-P pAbs (**b** and **e**) and with TpMab-1 antibody (**c** and **f**) in serial sections (**a-c** and **d-f**) prepared from the identical pancreatic cancer lesions. Scale bar: 50 μm.

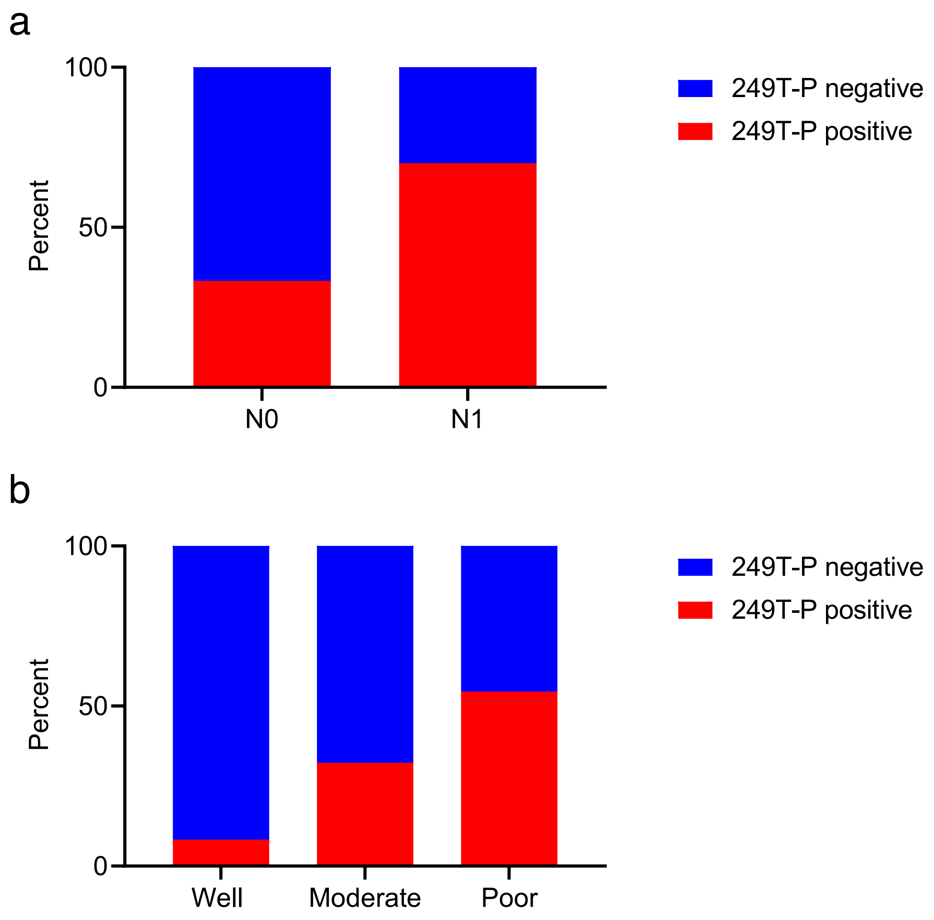

Supplementary Figure 6 Graphical representation for clinicopathological analysis of hTERT T249 phosphorylation
a, Lymph nodes metastasis was more frequently detected in pancreatic cancer with T249 phosphorylation (related to **Supplementary Table 2**). **b,** hTERT T249 phosphorylation correlated with the histological degree of differentiation in HCC (related to **Supplementary Table 3**).

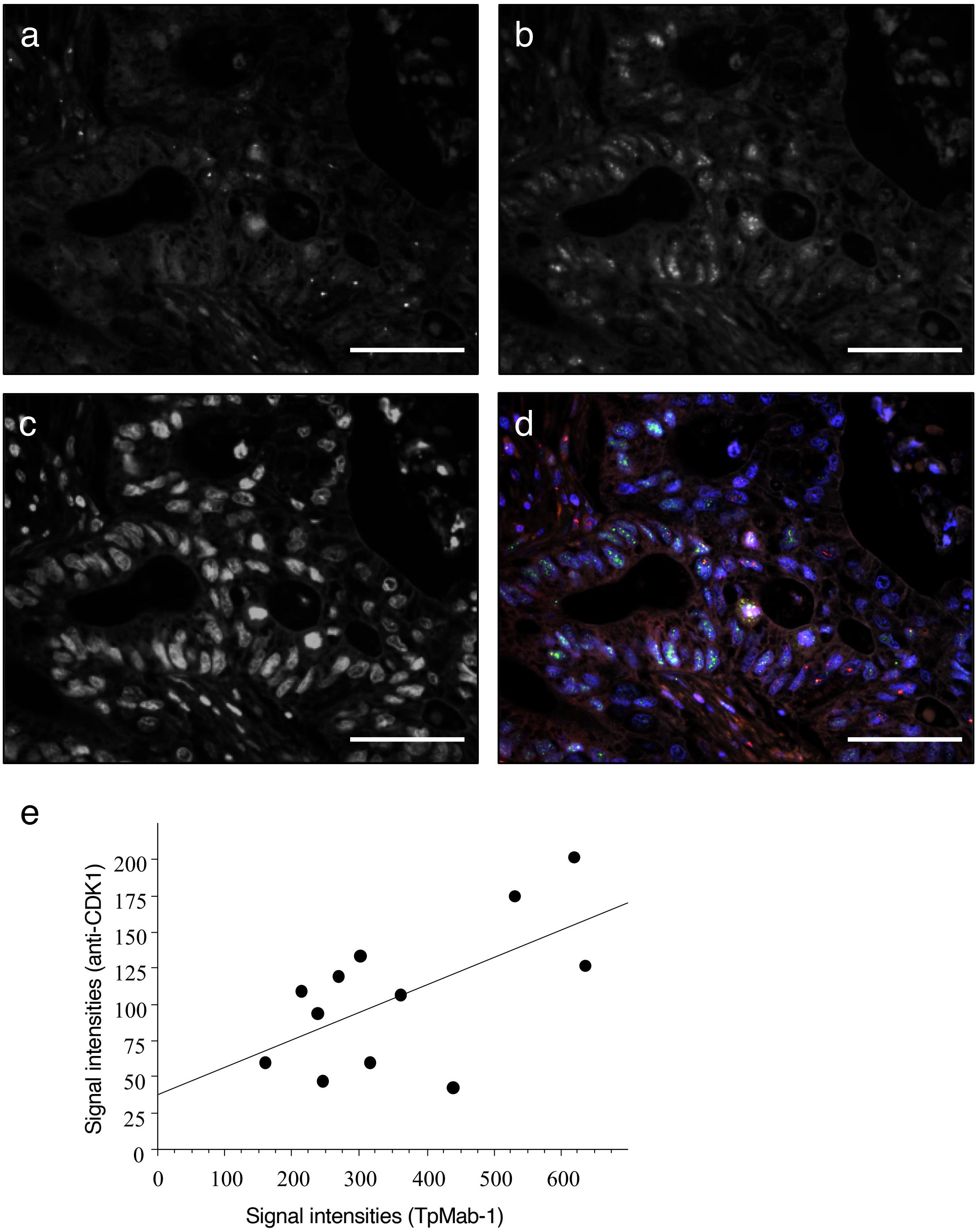

Supplementary Figure 7 Immunofluorescence images stained with anti-CDK1 and TpMab-1 antibodies

a-**c,** Representative immunofluorescence images stained with anti-CDK1 (**a**), TpMab-1 antibodies (**b**) and DAPI (**c**) prepared from the identical pancreatic cancer lesions. **d,** Merged image. Anti-CDK1 staining is shown in red, TpMab-1 in green and DAPI in blue. Scale bar: 50 μm. **e,** Scatter plot analysis of signal intensities between anti-CDK1 and TpMab-1 staining (linear regression analysis, p=0.0350).

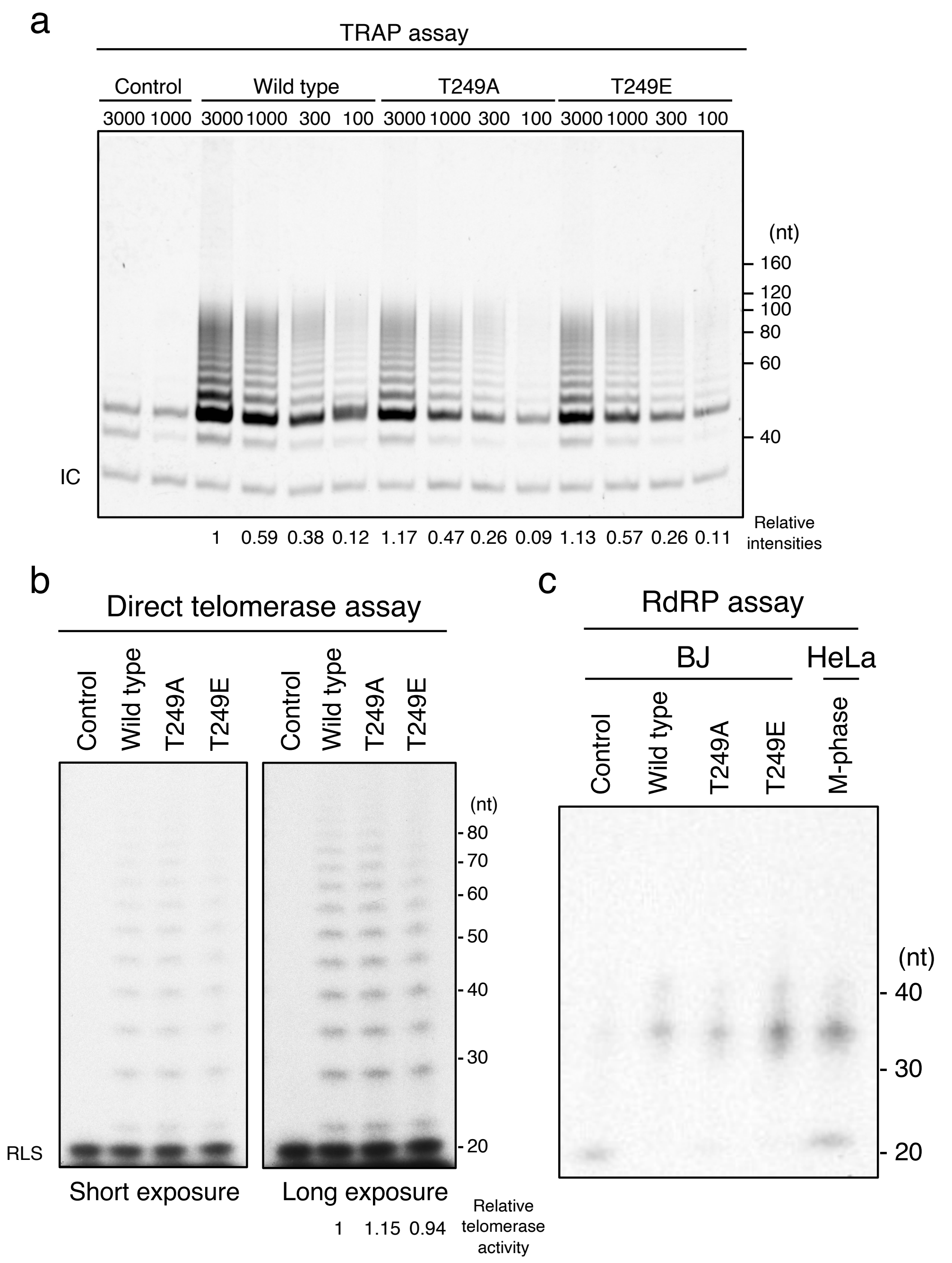

Supplementary Figure 8 Telomerase and RdRP activities of hTERT from BJ cells expressing wild type or mutant hTERT

a, TRAP assay using serial dilution of extracts from BJ cells ectopically expressing wild type or mutant hTERT (T249A, T249E). IC indicates internal control. The relative intensities are noted below the panel. **b,** Direct telomerase assay using hTERT proteins immunoprecipitated with anti-hTERT mouse mAb (clone 10E9-2) from BJ cells ectopically expressing wild type or mutant hTERT (T249A, T249E). RLS indicates recovery/loading standard. The relative telomerase activities are noted below the panel. **c,** RdRP assay using hTERT proteins immunoprecipitated with anti-hTERT mouse mAb (clone 10E9-2) from BJ cells ectopically expressing wild type or mutant hTERT (T249A, T249E). All experiments were repeated twice with similar results. Source data are provided in the Source Data file.

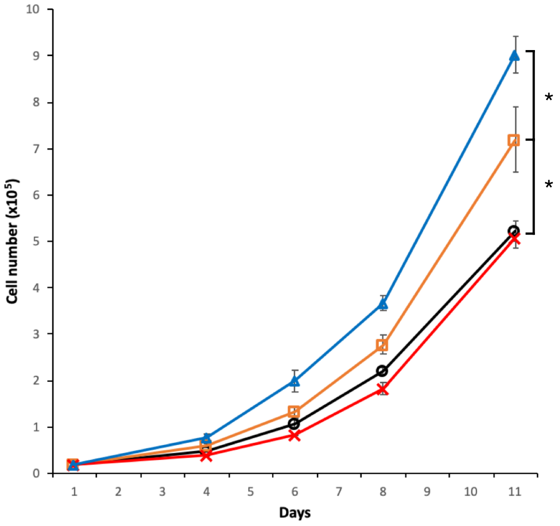

Supplementary Figure 9 Cell proliferation assay using Saos2 cells expressing wild type or mutant hTERT

Wild type hTERT (orange line), hTERT-T249A (red line), hTERT-T249E (blue line) or control retrovirus (black line) were stably infected in Saos2 cells. This assay was done in triplicate and data are shown as the mean ± SD. Asterisk indicates statistically significant values (p<0.05 by Student's t-test, two-sided).

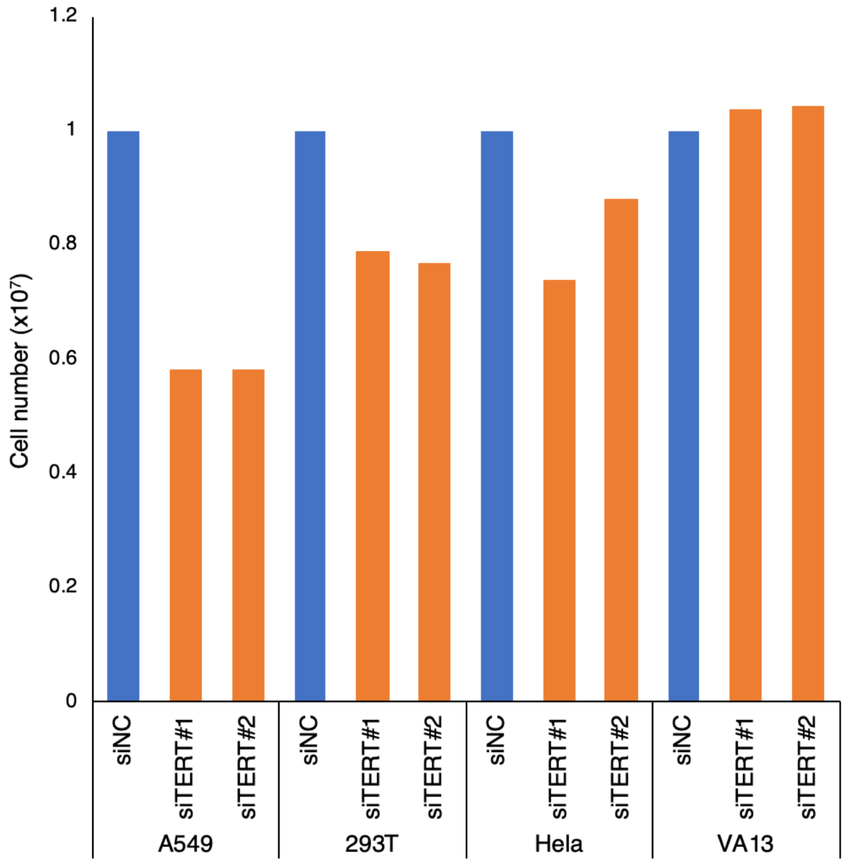

Supplementary Figure 10 Cell proliferation assay using cancer cell lines with suppression of hTERT
Cell proliferation assay using A549 (*n*=1), 293T (*n*=2), HeLa (*n*=2), VA13 (*n*=1) cells transfected with two different siRNAs specific for hTERT or siNC.

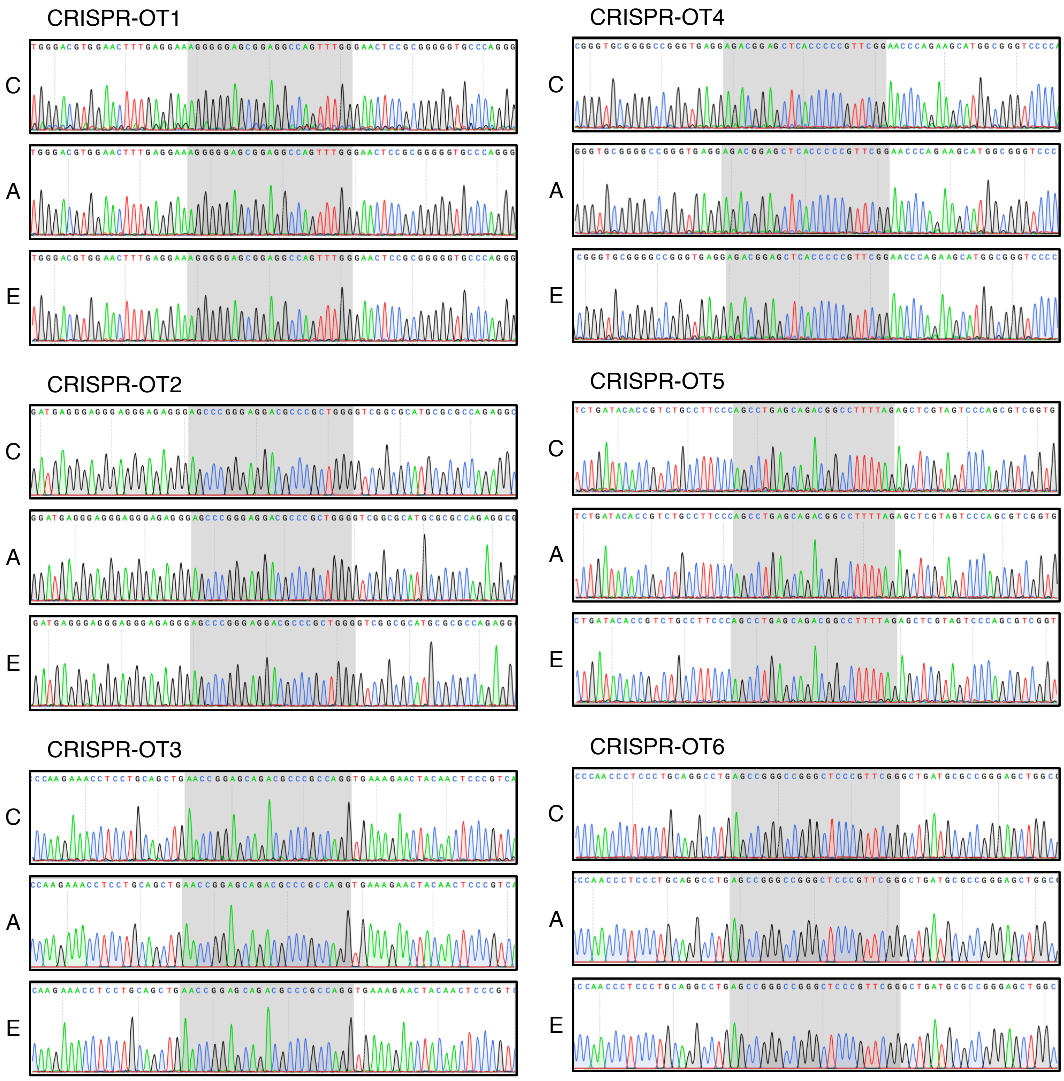

Supplementary Figure 11 Sanger Sequencing traces of off-target (OT) analysis for 293T-CRISPR cells

Six candidate OT mutation loci corresponding to the guide RNA were examined in Control-CRISPR, T249A-CRISPR and T249E-CRISPR cells (denoted as “C”, “A”, “E”). No OT mutations were detected.

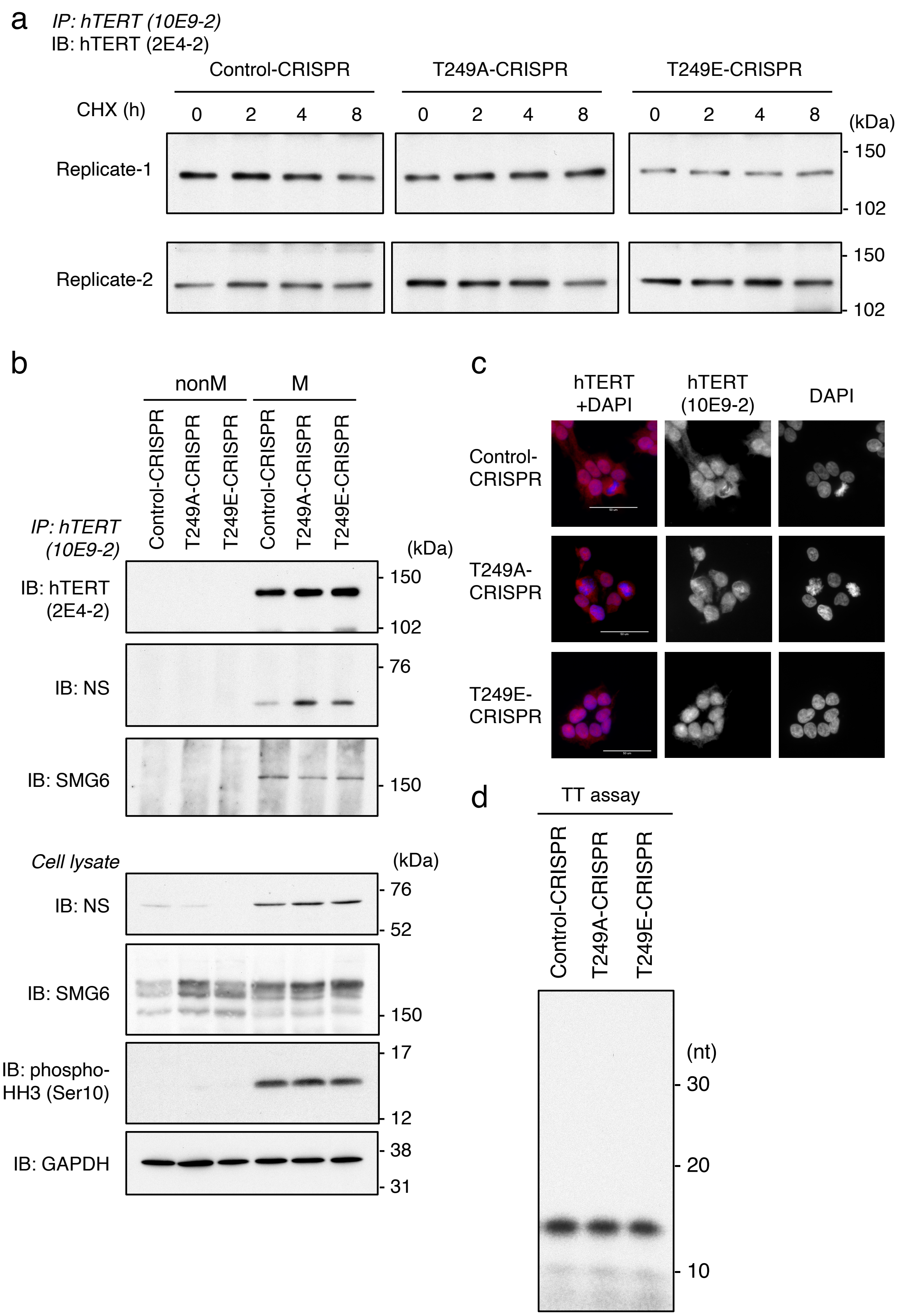
a
IP: hTERT (10E9-2)
IB: hTERT (2E4-2)
Control-CRISPR
T249A-CRISPR
T249E-CRISPR
CHX (h)
0 2 4 8
(kDa)
150
102
Replicate-1
Replicate-2
b
nonM
M
Control-CRISPR
T249A-CRISPR
T249E-CRISPR
IP: hTERT (10E9-2)
IB: hTERT (2E4-2)
IB: NS
IB: SMG6
Cell lysate
IB: phospho-HH3 (Ser10)
IB: GAPDH
76
52
17
12
38
31
c
hTERT +DAPI
hTERT (10E9-2)
DAPI
Control-CRISPR
T249A-CRISPR
T249E-CRISPR
d
TT assay
(nt)
30
20
10

Supplementary Figure 12 Examination of hTERT proteins by introducing substitutions at T249

a, 293T-CRISPR cells were synchronized to M-phase using nocodazole (100 ng/mL) and then treated with cycloheximide (CHX, 100 μg/mL) for 8 hours in duplicate. The endogenous hTERT proteins were immunoprecipitated with anti-hTERT mAb (clone 10E9-2) and detected by anti-hTERT mAb (clone 2E4-2). **b,** Immunoblotting using the indicated antibodies in 293T-CRISPR cells treated with DMSO (denoted as "non M") or nocodazole to manipulated cells in mitotic phase (denoted as "M"). hTERT complexes were immunoprecipitated with anti-hTERT mAb (clone 10E9-2) and immunoblotted with anti-hTERT mAb (clone 2E4-2), anti-nucleostemin (NS) rabbit pAbs (Bethyl, A300-600A) or anti-SMG6 rabbit pAbs. Cells arrested in mitosis with nocodazole were confirmed by anti-phospho-histone H3 (Ser10) antibodies. **c,** Immunostaining of 293T-CRISPR cells with anti-hTERT mAb (clone 10E9-2) followed by DAPI staining. Scale bar: 50 μm. **d,** IP-terminal transferase (TT) assay with oligo(C)14 primer using 293T-CRISPR cells synchronized to M-phase. Experiments were repeated twice (for **a**, **b**, **c**) and three times (for **d**) with similar results. Source data are provided in the Source Data file.

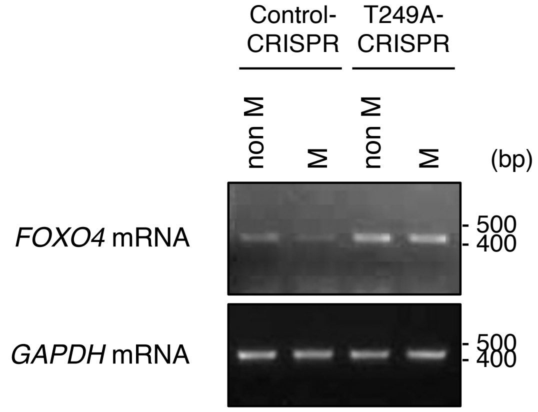

Supplementary Figure 13 Expression of *FOXO4* mRNAs in Control-CRISPR and T249A-CRISPR in mitosis
Control-CRISPR and T249A-CRISPR were manipulated in mitosis by nocodazole treatment. *FOXO4* mRNAs were detected by RT-PCR. Experiments were repeated twice with similar results. Source data are provided in the Source Data file.

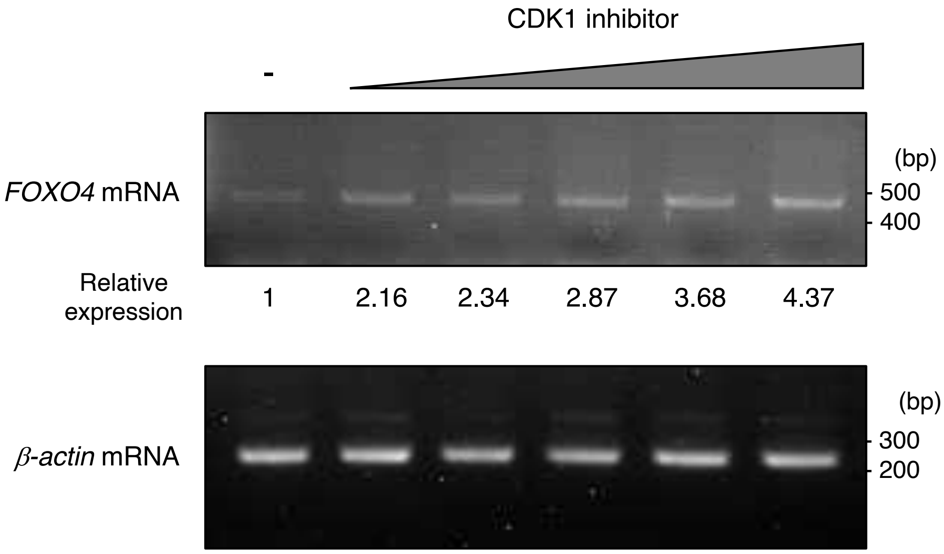

Supplementary Figure 14 Effects of inhibition of CDK1 activity on *FOXO4* expression

Increase of *FOXO4* mRNAs were detected by RT-PCR (upper panel). Total RNAs were extracted from HeLa cells treated with CDK1 inhibitor, RO-3306 (0, 0.15625, 0.3125, 0.625, 1.25, 2.5 μM) and nocodazole (100 ng/mL). *β-actin* was used as an internal control (lower panel). Experiments were repeated twice with similar results. Source data are provided in the Source Data file.

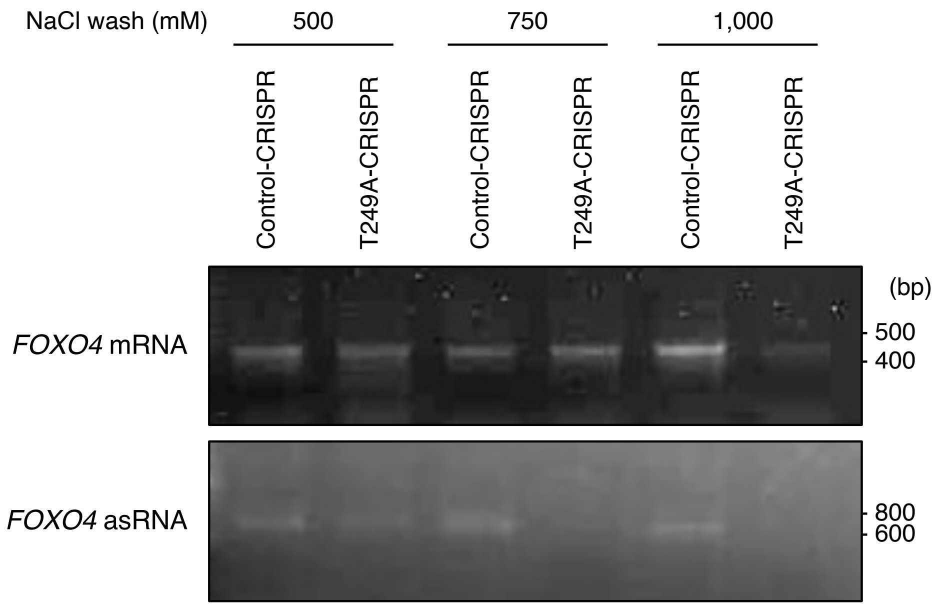

Supplementary Figure 15 Direct association between hTERT and *FOXO4* RNAs assessed by UV crosslinking

Control-CRISPR and T249A-CRISPR were UV-crosslinked. Immune complexes were isolated from the cell lysates with anti-hTERT mAb (clone 10E9-2) and washed under high salt conditions (500, 750 or 1,000 mM NaCl). Associated RNAs were subjected to RT-PCR. Experiments were repeated twice with similar results. Source data are provided in the Source Data file.

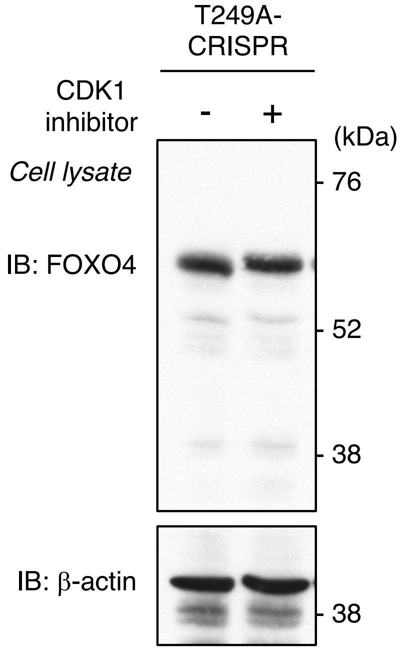

Supplementary Figure 16 Effects of inhibition of CDK1 activity on FOXO4 expression in T249A-CRISPR
Immunoblotting of FOXO4 in T249A-CRISPR treated with CDK1 inhibitor, RO-3306 (0.5 *μ*M). Experiments were repeated twice with similar results. Source data are provided in the Source Data file.

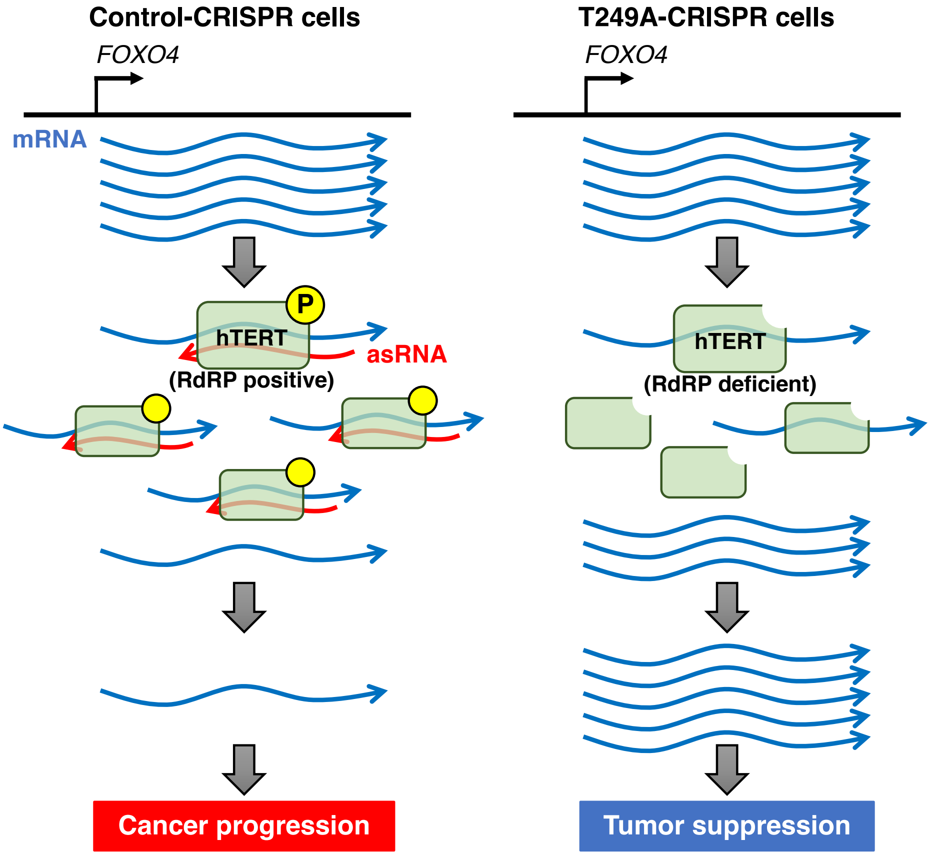

Supplementary Figure 17 A model for regulation of *FOXO4* expression via phosphorylation of hTERT at T249

In Control-CRISPR cells, hTERT proteins have phosphorylation-dependent RdRP activity and synthesize asRNAs from *FOXO4* mRNAs. These RNAs form double-stranded RNAs and might be degraded. In T249A-CRISPR cells, RdRP-deficient hTERT-T249A proteins interact with less *FOXO4* mRNAs. Without producing asRNA, more *FOXO4* mRNAs are retained in the T249A-CRISPR cells and increase of *FOXO4* expression cause tumor suppression.

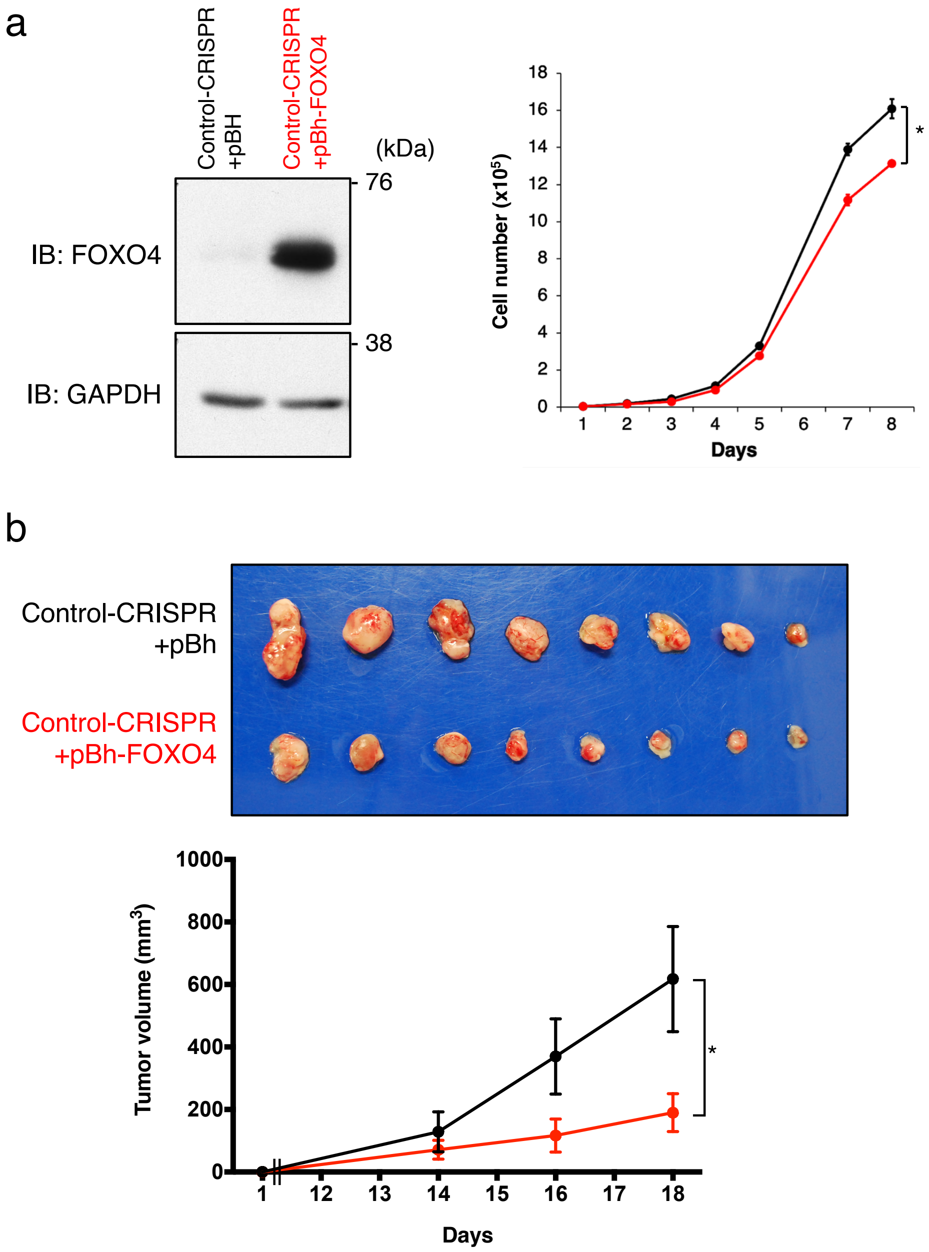
a
Control-CRISPR +pBH
Control-CRISPR +pBh-FOXO4
(kDa)
76
38
IB: FOXO4
IB: GAPDH
Cell number (x10^5)
Days
*
b
Control-CRISPR +pBh
Control-CRISPR +pBh-FOXO4
Tumor volume (mm^3)
Days
*

Supplementary Figure 18 Effects of FOXO4 upregulation in Control-CRISPR
a, Immunoblotting of FOXO4 in Control-CRISPR infected with pBh control and pBh-FOXO4 retroviruses (left panel). Experiments were repeated twice with similar results. Source data are provided in the Source Data file. Cell proliferation assay using Control-CRISPR+pBh (black line) or Control-CRISPR+pBh-FOXO4 (red line) (right panel). The assay was done in triplicate and data are shown as the mean ± SD. Asterisk indicates statistically significant values (p<0.05 by Student's t-test, two-sided). **b,** Tumor appearances generated from subcutaneous injection of 1x 10^6 cells of Control-CRISPR+pBh-FOXO4 or Control-CRISPR+pBh in eight NOD/SCID mice for each are represented (upper panel). The volume curves of tumors derived from mice with Control-CRISPR+pBh-FOXO4 (red line) or Control-CRISPR+pBh (black line) are demonstrated as the mean ± SEM (n=8) (lower panel). Asterisk indicates statistically significant values (p=0.0315 by unpaired t-test, two-sided).

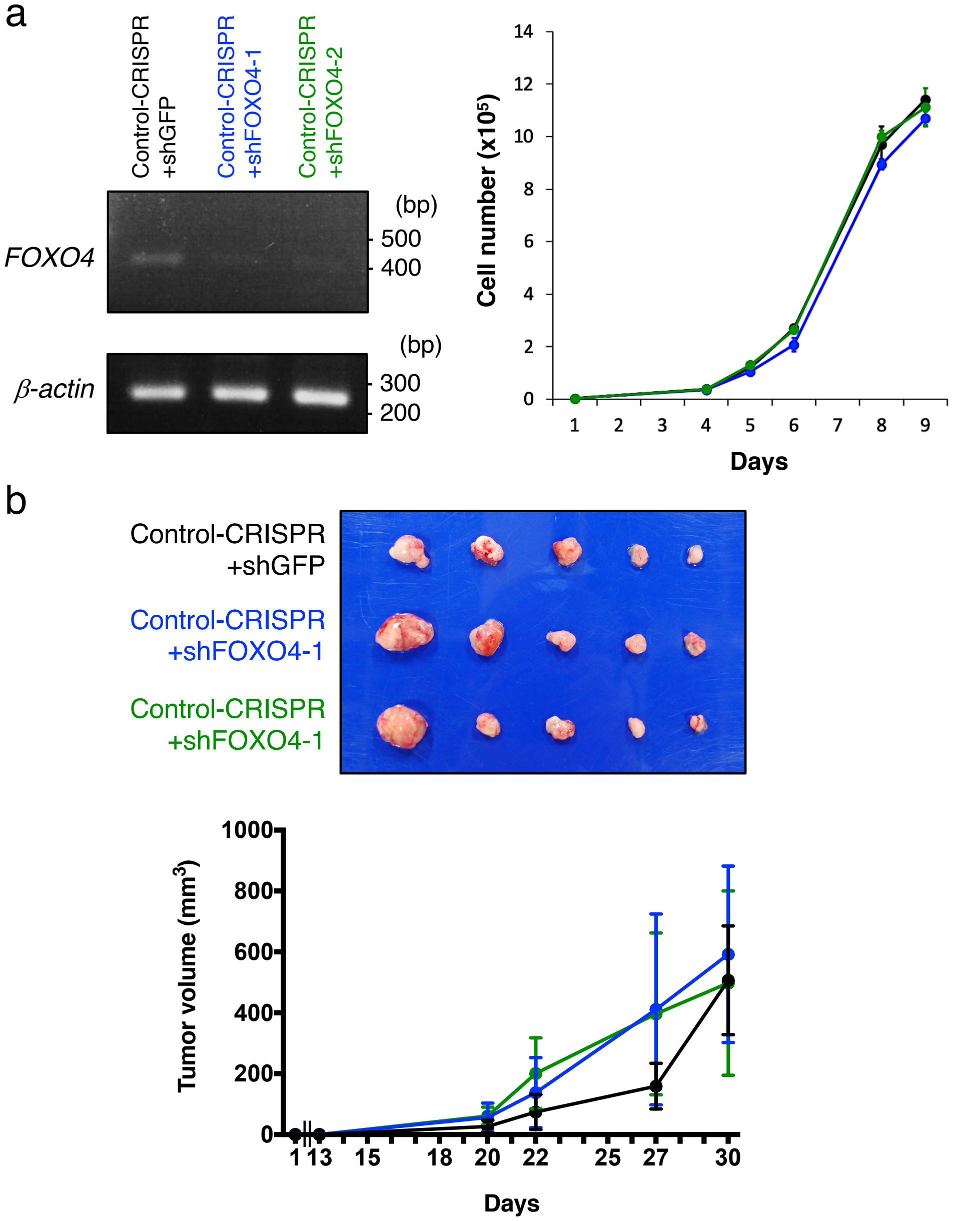
a
Control-CRISPR +shGFP
Control-CRISPR +shFOXO4-1
Control-CRISPR +shFOXO4-2
FOXO4
(bp)
500
400
β-actin
(bp)
300
200
Cell number (x10⁵)
Days
b
Control-CRISPR +shGFP
Control-CRISPR +shFOXO4-1
Control-CRISPR +shFOXO4-1
Tumor volume (mm³)
Days

Supplementary Figure 19 Effects of FOXO4 downregulation in Control-CRISPR

a, RT-PCR of *FOXO4* in Control-CRISPR expressing shFOXO4-1, 2 or shGFP control (left panel). Experiments were repeated twice with similar results. Source data are provided in the Source Data file. Cell proliferation assay using Control-CRISPR+shFOXO4-1 (blue line), Control-CRISPR+shFOXO4-2 (green line) or Control-CRISPR+shGFP (black line) (right panel). The assay was done in triplicate and data are shown as the mean ± SD. There was no statistically significant differences (by Student's t-test, two-sided). **b,** Tumor appearances generated from subcutaneous injection of 1x 10^6 cells of Control-CRISPR+shFOXO4-1, -2 or Control-CRISPR +shGFP in five NOD/SCID mice for each are represented (upper panel). The volume curves of tumors derived from mice with Control-CRISPR+shFOXO4-1 (blue line), Control-CRISPR+shFOXO4-2 (green line) or Control-CRISPR+shGFP (black line) are demonstrated as the mean ± SEM (n=5) (lower panel). There was no statistically significant differences (by unpaired t-test, two-sided).

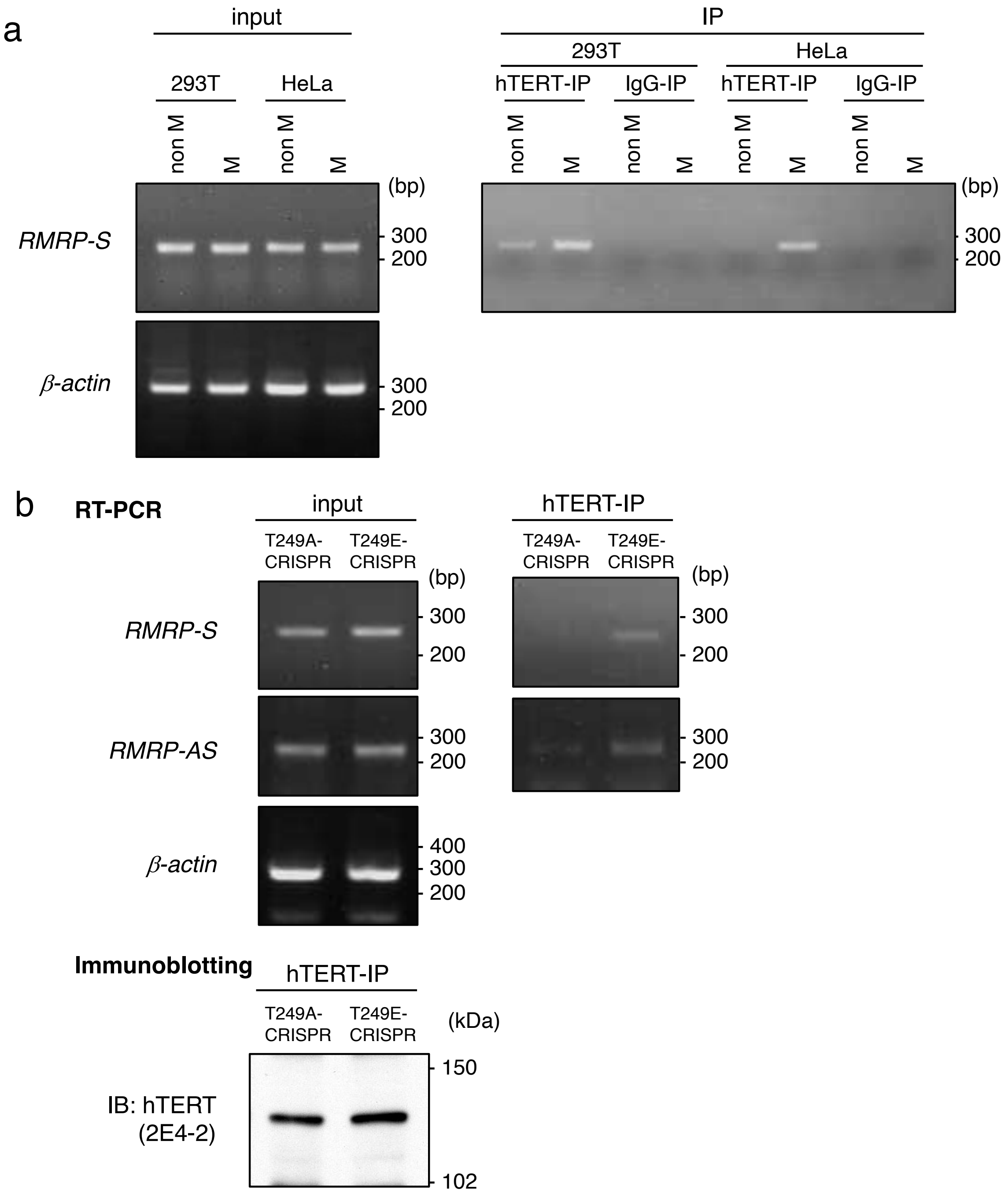

Supplementary Figure 20 Phosphorylation of hTERT at T249 affects the binding affinity between hTERT and *RMRP* RNAs

a, Immune complexes were isolated with anti-hTERT mAb (clone 10E9-2) from 293T and HeLa cells treated with DMSO (non M) or nocodazole (M). Associated RNAs were purified and subjected to RT-PCR. **b,** RNAs associated with hTERT mutant proteins were isolated from T249A-CRISPR and T249E-CRISPR cells, and subjected to RT-PCR (upper panels, RT-PCR). Similar expression of hTERT mutant proteins was confirmed by immunoblotting with anti-hTERT mAb (clone 2E4-2) (lower panel, immunoblotting). All experiments were repeated three times with similar results. Source data are provided in the Source Data file.

Supplementary Table 1, MS data of hTERT from *in vitro* kinase assay

hTERT.191-306_Phosphorylation by CDK1-CycB

Description	Score	Coverage	# Proteins	# Unique Peptides	# Peptides	# PSMs	# AAs	MW [kDa]	calc. pI
Telomerase reverse transcriptase OS=Homo sapiens GN=TERT PE=1 SV=1 - [TERT_HUMAN]	781.66	7.77	1	12	12	46	1132	126.9	10.52

A2	Sequence	# PSMs	# Proteins	# Protein Groups	Protein Group Accessions	1	ΔCn	phosphoRS Site Probabilities	q-Value	PEP	IonScore	Exp Value	Charge	MH+ [Da]	ΔM [ppm]	RT [min]	# Missed Cleavages
High	TPVGQGSWAHPGR	2	1	1	O14746		0.0000		0	0.0001284	68	1.11476E-06	2	1349.67143	0.34	13.45	0
High (hTERT_274S)	GFcVVsPARPAEEATSLEGALSGTR	3	1	1	O14746	C3(Carbamidomethyl); S6(Phospho)	0.0000	S(6): 100.0; T(15): 0.0; S(16): 0.0; S(22): 0.0; T(24): 0.0	0	0.00005126	61	7.83771E-06	3	2642.23880	4.03	31.58	0
High	EAGVPLGLPAPGAR	12	1	1	O14746		0.0000		0	0.0002435	59	3.52226E-06	2	1304.73235	0.11	25.07	0
High	GFcVVSPARPAEEATSLEGALSGTR	1	1	1	O14746	C3(Carbamidomethyl)	0.0000		0	0.0002302	48	0.000118526	3	2562.26200	0.07	28.50	0
High	GPSDRGFcVVSPARPAEEATSLEGALSGTR	2	1	1	O14746	C8(Carbamidomethyl)	0.0000		0	0.01813	44	0.000280259	4	3074.51333	5.58	27.51	1
High	RGAAPEPER	1	1	1	O14746		0.0000		0	0.02462	43	0.000298225	2	982.50737	0.86	7.16	1
**High (hTERT_249T)**	**GAAPEPERtPVGQGSWAHPGR**	**1**	**1**	**1**	**O14746**	**T9(Phospho)**	**0.0000**	**T(9): 100.0;** S(15): 0.0	**0**	**0.007542**	**42**	**0.000698864**	**4**	**2237.02480**	**-0.03**	**15.88**	**1**
High (hTERT_274S)	GPSDRGFcVVsPARPAEEATSLEGALSGTR	8	1	1	O14746	C8(Carbamidomethyl); S11(Phospho)	0.0000	S(3): 0.0; S(11): 100.0; T(20): 0.0; S(21): 0.0; S(27): 0.0; T(29): 0.0	0	0.0002018	42	0.000769782	4	3154.46694	1.41	29.31	1
**High (hTERT_249T)**	**tPVGQGSWAHPGR**	**1**	**1**	**1**	**O14746**	**T1(Phospho)T1(Phospho)**	**0.0000**	**T(1): 100.0;** S(7): 0.0	**0**	**0.09146**	**36**	**0.001734836**	**2**	**1429.63677**	**-0.37**	**15.27**	**0**
High	AWNHSVREAGVPLGLPAPGAR	4	1	1	O14746		0.0000		0	0.00304	29	0.003476626	3	2155.15390	0.86	22.50	1
High	SLPLPKRPR	3	1	1	O14746		0.0000		0	0.02031	23	0.004720304	3	1063.67411	0.54	12.70	1
High	EAGVPLGLPAPGARR	2	1	1	O14746		0.0000		0	0.00247	21	0.01783279	3	1460.83353	0.14	21.23	1
High (hTERT_206S)	AWNHsVREAGVPLGLPAPGAR	1	1	1	O14746	S5(Phospho)	0.0000	S(5): 100.0	0	0.1816	20	0.081900648	3	2235.11753	-0.38	24.55	1
High	GAAPEPERTPVGQGSWAHPGR	2	1	1	O14746		0.0000		0	0.0787	15	0.258115362	5	2157.05717	-0.64	15.74	1
High	LGcERAWNHSVR	1	1	1	O14746	C3(Carbamidomethyl)	0.0000		0	0.06023	10	0.749015362	3	1484.71708	-0.36	11.67	1
Medium	GAAPEPER	1	1	1	O14746		0.0000		0.022	0.2064	35	0.002318774	2	826.40520	-0.26	7.43	0
Medium	AWNHSVR	1	1	1	O14746		0.0000		0.028	0.2521	26	0.007735331	2	869.43804	0.40	7.98	0

hTERT.191-306_Phosphorylation by IKK2

Description	Score	Coverage	# Proteins	# Unique Peptides	# Peptides	# PSMs	# AAs	MW [kDa]	calc. pI
Telomerase reverse transcriptase OS=Homo sapiens GN=TERT PE=1 SV=1 - [TERT_HUMAN]	1019.70	7.33	1	9	9	75	1132	126.9	10.52

A2	Sequence	# PSMs	# Proteins	# Protein Groups	Protein Group Accessions	Modifications	ΔCn	phosphoRS Site Probabilities	q-Value	PEP	IonScore	Exp Value	Charge	MH+ [Da]	ΔM [ppm]	RT [min]	# Missed Cleavages
High	TPVGQGSWAHPGR	5	1	1	O14746		0.0000		0	1.392E-14	56	2.11052E-05	3	1349.66883	-1.59	13.96	0
High	GFcVVSPARPAEEATSLEGALSGTR	8	1	1	O14746	C3(Carbamidomethyl)	0.0000		0	6.228E-16	49	8.53574E-05	3	2562.27518	5.22	28.64	0
High (hTERT_206S)	AWNHsVREAGVPLGLPAPGAR	42	1	1	O14746	S5(Phospho)	0.0000	S(5): 100.0	0	4.6E-15	47	0.000146669	3	2235.11972	0.60	25.11	1
High	GPSDRGFcVVSPARPAEEATSLEGALSGTR	2	1	1	O14746	C8(Carbamidomethyl)	0.0000		0	3.66009E-16	47	0.000192597	4	3074.50063	1.46	27.01	1
High	RGAAPEPER	1	1	1	O14746		0.0000		0	1.862E-14	43	0.000298225	2	982.50682	0.30	7.16	1
High (hTERT_283T)	GPSDRGFcVVSPARPAEEAtSLEGALSGTR	2	1	1	O14746	C8(Carbamidomethyl); T20(Phospho)	0.0000	S(3): 0.0; S(11): 0.0; T(20): 50.0; S(21): 50.0; S(27): 0.0; T(29): 0.0	0	3.976E-11	39	0.001401446	4	3154.46181	-0.21	27.83	1
High	EAGVPLGLPAPGAR	5	1	1	O14746		0.0000		0	0.000004751	37	0.000625746	2	1304.73332	0.86	24.45	0
High	GAAPEPERTPVGQGSWAHPGR	5	1	1	O14746		0.0000		0	1.655E-07	32	0.005081198	4	2157.05922	0.32	15.96	1
High	EAGVPLGLPAPGARR	1	1	1	O14746		0.0000		0	1.789E-11	26	0.004211957	3	1460.83820	3.34	21.00	1
High	SLPLPKRPR	3	1	1	O14746		0.0000		0	0.008781	26	0.002666674	3	1063.67411	0.54	12.72	1
High (hTERT_274S)	GFcVVsPARPAEEATSLEGALSGTR	1	1	1	O14746	C3(Carbamidomethyl); S6(Phospho)	0.0000	S(6): 96.6; T(15): 1.1; S(16): 1.1; S(22): 1.1; T(24): 0.0	0	0.003605	12	0.599837122	3	2642.22965	0.57	28.26	0

Magenta letters denote peptides which contain threonine 249 phosphorylated by CDK1. Blue letters denote a peptide which contains threonine residue phosphorylated by IKK2.
Green letters denote peptides which contain serine residues phosphorylated by both CDK1 and IKK2.

Supplementary Table 2, Clinicopathological analysis of the hTERT T249 phosphorylation in pancreatic cancer

		249T-P		
		Positive (n=26)	Negative (n=21)	*p* value
Age (yr, mean ± SE)		74.8 ± 1.3	74.8 ± 1.7	0.9972
Gender	Female	17	8	0.0623
	Male	9	13	
Histological differentiation	Well	10	12	0.3221
	Moderate	13	7	
	Poor	0	1	
	Adenoaquamous carcinoma	3	1	
Primary tumor (T)	T0	1	0	0.4146
	T1	2	0	
	T2	1	1	
	T3	22	20	
Lymph nodes (N)	N0	6	18	<0.0001*
	N1	20	3	
Metastasis (M)	M0	25	20	0.8771
	M1	1	1	
3-year survival	Survival	2	5	0.0426*
	Dead	19	8	
	No data	5	8	
CDK1 staining	Positive	17	7	0.0289*
	Negative	9	14	

Asterisk indicates statistically significant values (p<0.05) examined with Chi square test.

Supplementary Table 3, Clinicopathological analysis of the hTERT T249 phosphorylation in liver cancer

		249T-P		
		Positive (n = 29)	Negative (n = 71)	*p* value
Age (yr, mean ± SE)		65.7 ± 2.2	64.8 ± 1.2	0.7155
Gender	Female	4	24	0.0432*
	Male	25	47	
Etiology	HBV	5	16	0.7889
	HCV	11	28	
	B+C	0	1	
	other	13	26	
AFP (ng/ml, mean ± SE)		60.5 ± 24.1	2,311 ± 1,033	0.165
Histological grade	Well	2	22	0.0122*
	Moderate	21	44	
	Poor	6	5	
Tumor size	<5cm	22	52	0.7862
	>5cm	7	19	
BCLC stage	A	15	46	0.3183
	B	7	16	
	C	7	9	
CDK1 staining	Positive	19	6	< 0.0001*
	Negative	10	65	

Asterisk indicates statistically significant values ($p<0.05$) examined with either Fisher's exact test or Chi square test.
Abbreviation: AFP, alphafetoprotein. BCLC, Barcelona Clinic Liver Cancer

Supplementary Table 4, Clinicopathological analysis of the CDK1 expression in liver cancer

		CDK1		
		Positive (n = 25)	Negative (n = 75)	*p* value
Age (yr, mean ± SE)		65.7 ± 2.1	64.8 ± 1.2	0.72
Gender	Female	6	22	0.8
	Male	19	53	
Etiology	HBV	3	18	0.56
	HCV	11	28	
	B+C	0	1	
	other	11	28	
AFP (ng/ml, mean ± SE)		62.0 ± 27.4	2,189 ± 978	0.21
Histological grade	Well	2	22	0.044*
	Moderate	18	47	
	Poor	5	6	
Tumor size	<5cm	19	55	0.99
	>5cm	6	20	
BCLC stage	A	12	49	0.27
	B	7	16	
	C	6	10	

Asterisk indicates statistically significant values (p<0.05) examined with either Fisher's exact test or Chi square test.
Abbreviation: AFP, alphafetoprotein. BCLC, Barcelona Clinic Liver Cancer

Supplementary Table 5, GO analysis of genes differentially expressed in T249A-CRISPR cells (FDR < 0.01).

UP/DOWN	Term	# of genes	*p* value	Fold Enrichment	FDR
UP	GO:0042254~ribosome biogenesis	82	1.87E-21	3.213667394	3.70E-18
	GO:0006364~rRNA processing	66	1.86E-17	3.221243362	3.67E-14
	GO:0016072~rRNA metabolic process	66	7.31E-17	3.139545161	2.22E-13
	GO:0034470~ncRNA processing	80	1.07E-14	2.555524484	2.11E-11
	GO:0006281~DNA repair	89	2.03E-12	2.204682314	4.02E-09
	GO:0008380~RNA splicing	73	4.61E-12	2.396043784	9.11E-09
	GO:0006412~translation	98	3.74E-11	2.007242885	7.38E-08
	GO:0006397~mRNA processing	78	1.09E-10	2.174230465	2.16E-07
	GO:0022618~ribonucleoprotein complex assembly	45	7.41E-10	2.786817531	1.46E-06
	**GO:0044770~cell cycle phase transition**	84	8.92E-10	2.016154645	1.76E-06
	GO:0016458~gene silencing	50	9.89E-10	2.594665422	1.96E-06
	GO:0071826~ribonucleoprotein complex subunit organization	46	1.23E-09	2.708225667	2.43E-06
	**GO:0044772~mitotic cell cycle phase transition**	79	2.89E-09	2.017882404	5.71E-06
	GO:0000375~RNA splicing, via transesterification reactions	55	3.24E-09	2.383153092	6.40E-06
DOWN	GO:0000184~nuclear-transcribed mRNA catabolic process, nonsense-mediated decay	36	1.65E-17	5.788847712	3.23E-14
	GO:0006614~SRP-dependent cotranslational protein targeting to membrane	32	2.11E-17	6.592854338	4.13E-14
	GO:0006613~cotranslational protein targeting to membrane	33	2.32E-17	6.33682116	4.53E-14
	GO:0006413~translational initiation	43	1.87E-16	4.499884707	4.33E-13
	GO:0045047~protein targeting to ER	32	2.71E-16	6.085711697	4.33E-13
	GO:0070972~protein localization to endoplasmic reticulum	35	5.86E-16	5.408200824	1.09E-12
	GO:0072599~establishment of protein localization to endoplasmic reticulum	32	9.89E-16	5.860314967	1.95E-12
	GO:0006612~protein targeting to membrane	39	2.17E-13	4.059810303	4.25E-10
	GO:0019080~viral gene expression	37	6.88E-12	3.811493914	1.35E-08
	GO:0019058~viral life cycle	61	7.93E-12	2.651631525	1.55E-08
	GO:0000956~nuclear-transcribed mRNA catabolic process	38	1.33E-11	3.648472789	2.60E-08
	GO:0006402~mRNA catabolic process	39	2.74E-11	3.49033465	5.36E-08
	GO:0044033~multi-organism metabolic process	38	4.24E-11	3.512081283	8.29E-08
	GO:0006401~RNA catabolic process	41	7.21E-11	3.256711179	1.41E-07
	GO:0019083~viral transcription	34	1.13E-10	3.71531018	2.21E-07
	GO:0006518~peptide metabolic process	86	1.45E-10	2.079408826	2.85E-07
	GO:0090150~establishment of protein localization to membrane	51	1.60E-10	2.741050853	3.13E-07
	GO:0043043~peptide biosynthetic process	74	3.21E-10	2.194323333	6.28E-07
	GO:0043604~amide biosynthetic process	79	3.54E-10	2.122970758	6.92E-07
	GO:0006412~translation	70	2.03E-09	2.159905477	3.97E-06

Supplementary Table 6, Putative phosphorylation sites of hTERT

TERT (human)		Kinase	LTP	HTP
T26-p	REVLPLA**t**FVRRLGP		0	1
Y122-p	FTTSVRS**y**LPNTVTD		0	1
S134-p	VTDALRG**s**GAWGLLL		0	1
S227-p	GARRRGG**s**ASRSLPL	Akt-1 (in vitro)	3	0
S679-p	RPGLLGA**s**VLGLDDI		0	1
T692-p	DIHRAWR**t**FVLRVRA		0	1
Y707-p	QDPPPEL**y**FVKVDVT	Src (putative)	5	0
T767-p	KSHVSTL**t**DLQPYMR		0	1
S824-p	AVRIRGK**s**YVQCQGI	Akt-1 (in vitro)	2	0
S948-p	EVQSDYS**s**yARTSIR		0	1
Y949-p	VQSDYSs**y**ARTSIRA		0	1
T1113-p	KLPGTTL**t**ALEAAAN		0	1
S1125-p	AANPALP**s**DFKTILD		0	1

Modified from PhosphoSitePlus (https://www.phosphosite.org)

LTP (Low Throughput Papers): The number of records in which this modification site was determined using methods other than discovery mass spectrometry.
HTP (High Throughput Papers): The number of records in which this modification site was assigned using ONLY proeomic discovery mass spectrometry.

Supplementary Table 7, Sequences of primers, siRNAs and oligos used in the work

Mutagenic primers

T249A	AGCCGGAGCGGGCGCCCGTTGGG
	CCCAACGGGCGCCCGCTCCGGCT
T249E	TGAGCCGGAGCGGGAGCCCGTTGGGCAG
	CTGCCCAACGGGCTCCCGCTCCGGCTCA

PCR primers for qPCR and RT-PCR

hTERT_F	CGGAAGAGTGTCTGGAGCAA
hTERT_R	GGATGAAGCGGAGTCTGGA
hLINE1_F	TTGGAAAACACTCTGCAGGATATTAT
hLINE1_R	TTGGCCTGCCTTGCTAGATT
hGAPDH_F	GAAGGTGAAGGTCGGAGTCA
hGAPDH_R	GAAGATGGTGATGGGATTTC
Alpha-satellite_RT	CCGTAAAACGACGGCCAGCTTCTGTCTAGTTTTTATGTGAAGATA
Alpha-satellite_F	CATTCTCAGAAACTTCTTTGTGATGTG
Alpha-satellite_R	CCGTAAAACGACGGCCAG
RMRP-S_RT	AGCCGCGCTGAGAATGAG
RMRP-AS_RT	GTGCTGAAGGCCTGTATCCT
RMRP_F	TGCTGAAGGCCTGTATCCT
RMRP_R	TGAGAATGAGCCCCGTGT
β-actin_F	CAAGAGATGGCCACGGCTGCT
β-actin_R	TCCTTCTGCATCCTGTCGGCA
FOXO4_RT	AAGTGTCAGTCGCTTCTCCG
FOXO4_F	AAAAAGTGCTTCGCTCGGC
FOXO4_R	GCTGGTTAGCGATCTCTGGT
FOXO4 asRNA_RT	GGGATACAGTGCCTCAGGTTT
FOXO4 asRNA_F	AAGTGTCAGTCGCTTCTCCG
FOXO4 asRNA_R	AAAAAGTGTCTTCGCTCGGC
RNaseP_F	GTCACTCCACTCCCATGTCC
RNaseP_R	AATTGGGTTATGAGGTCCCC

siRNAs

TERT siRNA #1	GUGUCUGUGCCCGGGAGAATT
	UUCUCCCGGGCACAGACACTT
TERT siRNA #2	GCAUUGGAAUCAGACAGCATT
	UGCUGUCUGAUUCCAAUGCTT

Oligos for CRISPR

guide RNA sequence	AGCCGGAGCGGacgCCCGTT GGG
Insert sequence of donor plasmid (pBluescript/SalI-NotI) for T249A	gtcgacGTCCCCCTGGGCCTGCCAGCCCCGGGTGCGAGGAGGCGCGGGGGCAGTGCC AGCCGAAGTCTGCCGTTGCCCAAGAGGCCCAGGCGTGGCGCTGCCCCTGAGCCGGA GCGGgccCCCGTTGGGCAGGGGTCCTGGGCCCACCCGGGCAGGACGCGTGGACCGA GTGACCGTGGTTTCTGTGTGGTGTCACCTGCCAGACCCGCCGAAGAAGCCACCTCTT TGGcggccgc
Donor oligo for T249E	GCCGAAGTCTGCCGTTGCCCAAGAGGCCCAGGCGTGGCGCTGCCCCTGAGCCGGA GCGGgaaCCgGTTGGGCAGGGGTCCTGGGCCCACCCGGGCAGGACGCGTGGACCGA GTGACCGTG
CRISPR PCR_F	AGCTACCTGCCCAACACGGT
CRISPR PCR_R for T249A	CGCACGCTCATCTTCCACGT
CRISPR PCR_R for T249E	CACACAGAAACCACGGTCAC

CRISPR OT sites

OT1	AGGGGGAGCGGAGGCCAGTTTGG
OT2	AGCCCGGGAGGACGCCCGCTGGG
OT3	AACCGGAGCAGACGCCCGCCAGG
OT4	AGACGGAGCTCACCCCCGTTCGG
OT5	AGCCTGAGCAGACGGCCTTTTAG
OT6	AGCCGGGCCGGGCTCCCGTTCGG
OT1 PCR_F	GTGGGAAGGTGACCCTGG
OT1 PCR_R	CACTTCAATCATGGCAGCT
OT2 PCR_F	CAGGACTCGGCCTACCTC
OT2 PCR_R	CCTCCAGTTGTCTGTGTCAAG
OT3 PCR_F	AAGCACGTAGCTCAGTGCAC
OT3 PCR_R	CTCACAGATCCAGGAGCTGA
OT4 PCR_F	TGCGGCTGAGAGATGGA
OT4 PCR_R	GCTCCAGTTCCTCCGACC
OT5 PCR_F	CCACAGTGCTGCACTGAG
OT5 PCR_R	GAGCCCAGAAGCAGCAC
OT6 PCR_F	GCATATCTGGTGTAGCCCG
OT6 PCR_R	CCCAGCACCTATTAAGTGCTC

FOXO4 shRNAs

shFOXO4-1 (TRCN0000010291)	CCGGCACTTAGGCTTTGTAGCAAGACTCGAGTCTTGCTACAAAGCCTAAGTGTTTTTG
shFOXO4-2 (TRCN0000039720)	CCGGCCAGCTTCAGTCAGCAGTTATCTCGAGATAACTGCTGACTGAAGCTGGTTTTTG

PCR primers for synthesis of FOXO4-asRNA

FOXO4-E1_F	TGCCGCGATCATAGACCTAG
FOXO4-E1_R_T7	TGTAATACGACTCACTATAGGGTTCAGCATCCACCAAGA
Luc_F	AAACGCTTCCACCTACCAGG
Luc_R_T7	TGTAATACGACTCACTATAGGGTCCTTTAGGCACCTCGTCC
