# Supplementary material for: CDK1 dependent phosphorylation of hTERT contributes to cancer progression
Source: Nat Commun. 2020 Mar 25;11:1557. doi: 10.1038/s41467-020-15289-7 (PMC7096428; doi:10.1038/s41467-020-15289-7)
Supplement: Supplementary file 3 — Description of Additional Supplementary Files [file 41467_2020_15289_MOESM3_ESM.pdf]

Description of Additional Supplementary Files

File Name: Supplementary Data 1
Description: MS data of hTERT isolated from 293T or HeLa cells synchronized to mitotic phase.
